# Heterologous protection elicited by candidate monomeric recombinant HIV-1 gp120 vaccine in the absence of cross neutralising antibodies in a macaque model

**DOI:** 10.1186/1742-4690-9-56

**Published:** 2012-07-16

**Authors:** Mark Page, Richard Stebbings, Neil Berry, Robin Hull, Deborah Ferguson, Leanne Davis, Laura Duffy, William Elsley, Joanna Hall, Claire Ham, Mark Hassall, Bo Li, Edward T Mee, Ruby Quartey-Papafio, Nicola J Rose, Nathalie Mathy, Gerald Voss, E James Stott, Neil Almond

**Affiliations:** 1 Division of Retrovirology, HPA-NIBSC, Blanche Lane, South Mimms, Potters Bar, Hertfordshire, EN6 3QG, UK; 2 Divisions of Biotherapeutics, HPA-NIBSC, Blanche Lane, South Mimms, Potters Bar, Hertfordshire, EN6 3QG, UK; 3 Division of Virology, HPA-NIBSC, Blanche Lane, South Mimms, Potters Bar, Hertfordshire, EN6 3QG, UK; 4 GlaxoSmithKline Biologicals, Rue de l'Institut 89, B-1330, Rixensart, Belgium

**Keywords:** Envelope HIV-1 vaccine, Recombinant gp120, Macaque model, SHIV, Heterologous challenge, Protection, Cynomolgus macaque

## Abstract

**Background:**

Current data suggest that an efficacious human immunodeficiency virus type 1 (HIV-1) vaccine should elicit both adaptive humoral and cell mediated immune responses. Such a vaccine will also need to protect against infection from a range of heterologous viral variants. Here we have developed a simian-human immunodeficiency virus (SHIV) based model in cynomolgus macaques to investigate the breadth of protection conferred by HIV-1_W61D_ recombinant gp120 vaccination against SHIV_sbg_ and SHIV_SF33_ challenge, and to identify correlates of protection.

**Results:**

High titres of anti-envelope antibodies were detected in all vaccinees. The antibodies reacted with both the homologous HIV-1_W61D_ and heterologous HIV-1_IIIB_ envelope rgp120 which has an identical sequence to the SHIV_sbg_ challenge virus. Significant titres of virus neutralising antibodies were detected against SHIV_W61D_ expressing an envelope homologous with the vaccine, but only limited cross neutralisation against SHIV_sbg_, SHIV-4 and SHIV_SF33_ was observed. Protection against SHIV_sbg_ infection was observed in vaccinated animals but none was observed against SHIV_SF33_ challenge. Transfer of immune sera from vaccinated macaques to naive recipients did not confer protection against SHIV_sbg_ challenge. In a follow-up study, T cell proliferative responses detected after immunisation with the same vaccine against a single peptide present in the second conserved region 2 of HIV-1 _W61D_ and HIV-1 IIIB gp120, but not SF33 gp120.

**Conclusions:**

Following extended vaccination with a HIV-1 rgp120 vaccine, protection was observed against heterologous virus challenge with SHIV_sbg_, but not SHIV_SF33_. Protection did not correlate with serological responses generated by vaccination, but might be associated with T cell proliferative responses against an epitope in the second constant region of HIV-1 gp120. Broader protection may be obtained with recombinant HIV-1 envelope based vaccines formulated with adjuvants that generate proliferative T cell responses in addition to broadly neutralising antibodies.

## Background

In spite of significant efforts to make effective anti-retroviral therapies available to HIV-1 infected individuals, the development of an effective vaccine must be considered a priority that is likely to be the best hope for reducing the spread of HIV infection [1-3]. Recombinant HIV-1 envelope based AIDS vaccines have progressed to phase 3 trials [4-6] but they failed to demonstrate any evidence that the vaccine formulations tested (bivalent gp120 envelope antigen adjuvanted with Alum (AIDSVAX® B/E) were able to prevent acquisition of infection. However, in a recent phase 3 trial, where the AIDVAX B/E vaccine was used in prime-boost regimen with the canarypox ALVAC-HIV vCP1521 vaccine, modest efficacy against HIV-1 acquisition was demonstrated [7]. It is believed that a central problem with these vaccine formulations is that antibodies elicited are focused on the hypervariable regions of the envelope protein of HIV-1 which limits their ability to neutralise viruses expressing heterologous envelopes. Therefore, it is essential that we identify adaptive immune responses elicited by vaccination that have the potential to increase the breadth of protection with HIV-1 envelope based vaccines.

The experimental infection of macaques with defined SIV/HIV-1 (SHIV) chimeric viruses provides a model to establish the breadth of protection of candidate vaccines and characterise the key vaccine responses that correlate with the protection observed in a highly controlled manner which cannot be done readily in clinical trials [8]. In previous reports, we have utilised SHIV’s to study the protection conferred by HIV-1_W61D_ r (recombinant) gp120 formulated in a potent adjuvant in the macaque model. Three immunisations with HIV-1_W61D_ rgp120 in SBN1 adjuvant did not protect against challenge with heterologous SHIV_SF33_ that exhibits 85% amino acid identity across rgp120 [9]. Mooij *et al*. [10] reported however, that 5 immunisations with this vaccine protected macaques against detectable infection following challenge with the homologous SHIV_W61D_[11]. Furthermore, following an additional single boost of HIV-1_W61D_ rgp120 [12], these same animals were protected against challenge with heterologous SHIV_SF13_ and SHIV_HAN-2_ that shared overall 88% [13] and 82% [14] amino acid identity respectively across rgp120. A further single immunisation was given to these animals and challenged with a pathogenic SHIV_89.6P,_ but only one of the vaccinees was protected. In another study, Voss and colleagues [15] reported that whilst immunisation with AS02_A_–adjuvanted HIV-1_W61D_ rgp120 did not prevent SHIV_89.6p_ infection, it did prevent CD4+ cell decline and delayed the onset of AIDS. Thus, whilst protection against heterologous SHIV challenge is possible, the factors that conferred protection against selected heterologous SHIVs are poorly understood. Neutralising antibodies are associated with protection against homologous [16] and heterologous SHIV challenge [17]. However, Ellenberger et al. [18] could not determine an immune correlate of the protection conferred by a multi-protein DNA/MVA HIV-1 vaccine in rhesus monkeys against a highly heterologous SHIVSF162P3. Recent studies have also questioned the role of neutralising antibodies alone, since vaccine protection was observed against neutralising antibody resistant virus challenge [19] and with persistence of T cell responses [20].

This report investigates the potential of an extended immunisation protocol using the AS02_A_–adjuvanted HIV-1_W61D_ envelope vaccine to protect against heterologous SHIV challenges. Although protection against SHIV_SF33_ acquisition was not elicited by this vaccine regimen, SHIV_sbg_ challenge was resisted in the absence of detectable cross-neutralising antibodies. Furthermore, protection against SHIV_sbg_ was not transferred with immune serum. Since there is no evidence that this vaccine elicits CD8+ T cells responses, these observations raise the possibility that CD4+ T cells may be involved in the protection observed. Analysis of the CD4+ T cell responses elicited by this vaccine indicates that reactivity to a peptide in the C (constant) region 2 of HIV-1 gp120 hints at an association with the protection observed against SHIV challenge. Further studies using the SHIV/macaque model could establish whether these CD4+ cell responses to vaccination might provide effective anti-viral immunity directly.

## Results

### Antibody responses following immunisation with HIV-1_W61D_ rgp120

Eight macaques (A71-A78) were immunised with the vaccine and then split into two groups of four for subsequent challenge with either SHIV_sbg_ (group A; Table 1) or SHIV_SF33_ (group B; Table 1). All vaccinees seroconverted to HIV-1_W61D_ rgp120 as determined by ELISA following the second immunisation. Two weeks after the third immunisation, the mean log_10_ end point titre was 4.28+/−0.17. Five subsequent boosts failed to increase binding titres. On the day of challenge the mean log_10_ end point titre was 4.03+/−0.15.

**Table 1:** Immunisation schedule

**Group**	**Animal numbers**	**Vaccine**	**Schedule (weeks)**	**Challenge virus**
A	A71, A75, A76, A78	W61D rgp120+ AS02_A_	1, 4, 12, 20, 28, 36, 44, 86	SHIV_sbg_^a^
B	A72, A73, A74, A77	W61D rgp120+ AS02_A_	1, 4, 12, 20, 28, 36, 44, 92	SHIV_SF33_^b^
C	B234-B237	AS02_A_		SHIV_sbg_^c^
D	B238-B241	AS02_A_		SHIV_SF33_^d^
E	C37-C40	Serum transfer		SHIV_sbg_^e^
F	C41-C44	None		SHIV_sbg_^f^
G	G19; G21	W61D rgp120+ AS02_A_	1, 4, 8	None

^a^ = 10 MID_50_ SHIV_sbg_ challenge dose + 4 weeks after last immunisation.

^b^ = 50 MID_50_ SHIV_sbg_ challenge dose + 4 weeks after last immunisation.

^c^ = 10MID_50_ challenge dose contemporaneous with group A.

^d^ = 50MID_50_ challenge dose contemporaneous with group B.

^e^ = 50MID_50_ challenge dose given 24 hours following serum transfer.

^f^ = 50MID_50_ contemporaneous with group F.

To determine whether immunisation with HIV-1_W61D_ rgp120 elicited antibodies that cross react with the SHIV_sbg_ challenge virus, ELISAs were carried out using HIV-1_IIIB_ rgp120 (CFAR EVA607), since this isolate has an identical envelope sequence [21]. ELISA could not be performed against SHIV_SF33_ due to the lack of availability of SF33 rgp120.

Sera collected from all vaccinees were tested for their ability to neutralise homologous virus (SHIV_W61D_) and heterologous challenge viruses (SHIV_sbg_ and SHIV_SF33_). All vaccinees developed moderate titres (50% log _10_ titre >2.3) of neutralising antibodies against the homologous SHIV_W61D_ at either the day of challenge (group A) or after 7 immunisations (group B) (Table 2).

**Table 2:** **Serological responses following immunisation with HIV-1**_**W61D**_**rgp120**

**GROUP**	**ID NO.**	**VACCINE**	**SEROLOGICAL RESPONSES**							
			**Binding Antibodies (log_10_ reciprocal titre)**		**Virus Neutralisation Activity (50% virus inhibition log_10_ titre)**					
			**W61D rgp120 week 50**^**a**^	**IIIB rgp120 DOC**^**a**^	**SHIV**_**W61D**_**DOC**^**a**^	**SHIV**_**W61D**_ **week 50**^**a**^	**SHIV**_**sbg**_**DOC**^**a**^	**SHIV-4 DOC**^**a**^	**SHIV**_**SF33**_**week 50**^**a**^	**SHIV**_**SF33**_**week140**^**a**^
A	A71	30μg W61D rgp120/AS02_A_ i.m	3.9	3.5	2.9		<1	1.6		
	A75		3.9	3.7	3.1		<1	1.5		
	A76		4.2	3.8	3.1		<1	1.2		
	A78		4.1	3.5	3.2		<1	1.4		
C	B234	AS02_A_ i.m					<1			
	B235						<1			
	B236						<1			
	B237						<1			
B	A72	30μg W61D rgp120/AS02_A_ i.m	4.0			2.7		1.5	1.5	
	A73		3.9			2.7		1.4	1.4	
	A74		4.0			2.7		1.5	1.5	
	A77		4.3			2.3		1.6	1.6	
D	B238	AS02_A_ i.m								2.4
	B239									>2.7
										B240									2.2
	B241									>3.4									

DOC = day of challenge.

^a^ = time of sampling.

SHIV_sbg_ was refractory to neutralisation by both vaccine (group A) and infected (group C) naive control sera using both single cell infectivity and T cell line based neutralisation assays (Table 2). We also tested the ability of the sera to neutralise SHIV-4 [22], which has an identical envelope sequence to SHIV_sbg_, to examine whether the lack of neutralisation was a specific feature of the challenge virus. Very low titre (50% log _10_ titre <1.6) neutralising activity was observed in the vaccinees of group A and B against SHIV-4 (Table 2).

Low titres (50% log _10_ titre; range 1.5-1.6) of SHIV_SF33_ neutralisation were detected in group B vaccinees after 7 immunisations (Table 2). In infected controls (group D) at termination of the study higher titres (50% log _10_ titre; range 2.4-2.7) were observed (Table 2).

### Immunisation protects against challenge with heterologous SHIV_sbg_

**Figure 1:**
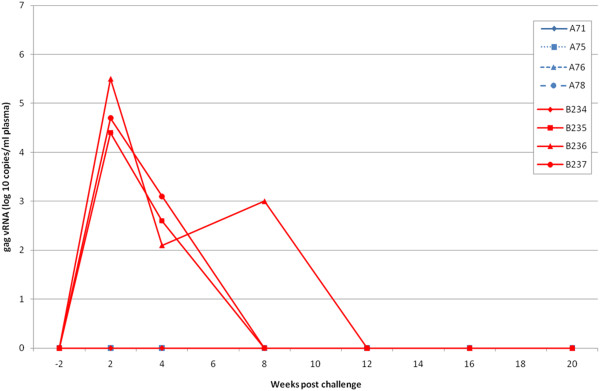
**Viral RNA levels in vaccinated macaques (A71, A75, A76 and A78; group A: blue lines) and contemporaneous controls given ASO2**_**A**_**adjuvant only (B234-237; group C: red lines) after challenge with 10MID**_**50**_**SHIV**_**sbg**_**and followed for twenty weeks.**

Four weeks after the 8^th^ immunisation, group A was challenged intravenously with 10 MID_50_ SHIV_sbg_ along with 4 naïve challenge controls (group C; B234-B237: Table 1). Following challenge, three of the controls became persistently infected with virus as determined by detection of proviral DNA, recovery of virus by co-culture (Table 3) and detection of viral RNA in plasma (Figure 1). Peak viral loads at 2 weeks were 5.3 +/− 0.6 mean log_10_ titre. By contrast, there was no evidence of detectable infection of the 4 vaccinees at any time point analysed after challenge (Table 3; Figure 1).

**Table 3:** **Detection of virus following challenge with heterologous SHIV**_**sbg**_**(10 MID50 dose)**

**GROUP**	**ID NO.**	**VACCINE**	**OUTCOME OF CHALLENGE(Weeks Post Challenge)**								
			**−4**	**2**		**4**		**8**	**12**	**16**	**20**
			**VI**	VT*	**PCR**	**VI**	**PCR**	**PCR**	**VI**	**PCR**	**VI**
A	A71	30 μg W61D rgp120/AS02_A_ i.m	-	<0.5	-	-	-	-	-	-	-
	A75		-	<0.5	-	-	-	-	-	-	-
	A76		-	<0.5	-	-	-	-	-	-	-
	A78		-	<0.5	-	-	-	-	-	-	-
C	B234	AS02_A_ i.m	-	<0.5	-	-	-	-	-	-	-
	B235		-	1.5	+	+	+	+	-	-	-
	B236		-	3.0	+	+	+	+	-	+	-
	B237		-	3.0	+	+	+	+	+	-	-

VT* = Virus Titration; Number of SIV + ve cells per 10^6^ PBMC.

VI = Virus isolation by co-culture of 5 x 10^6^ PBMC with 10^5^ C8166 indicator cells.

PCR = Diagnostic DNA PCR specific for SIV gag.

### Transfer of immune serum does not protect naïve macaques against challenge with SHIV_sbg_

**Figure 2:**
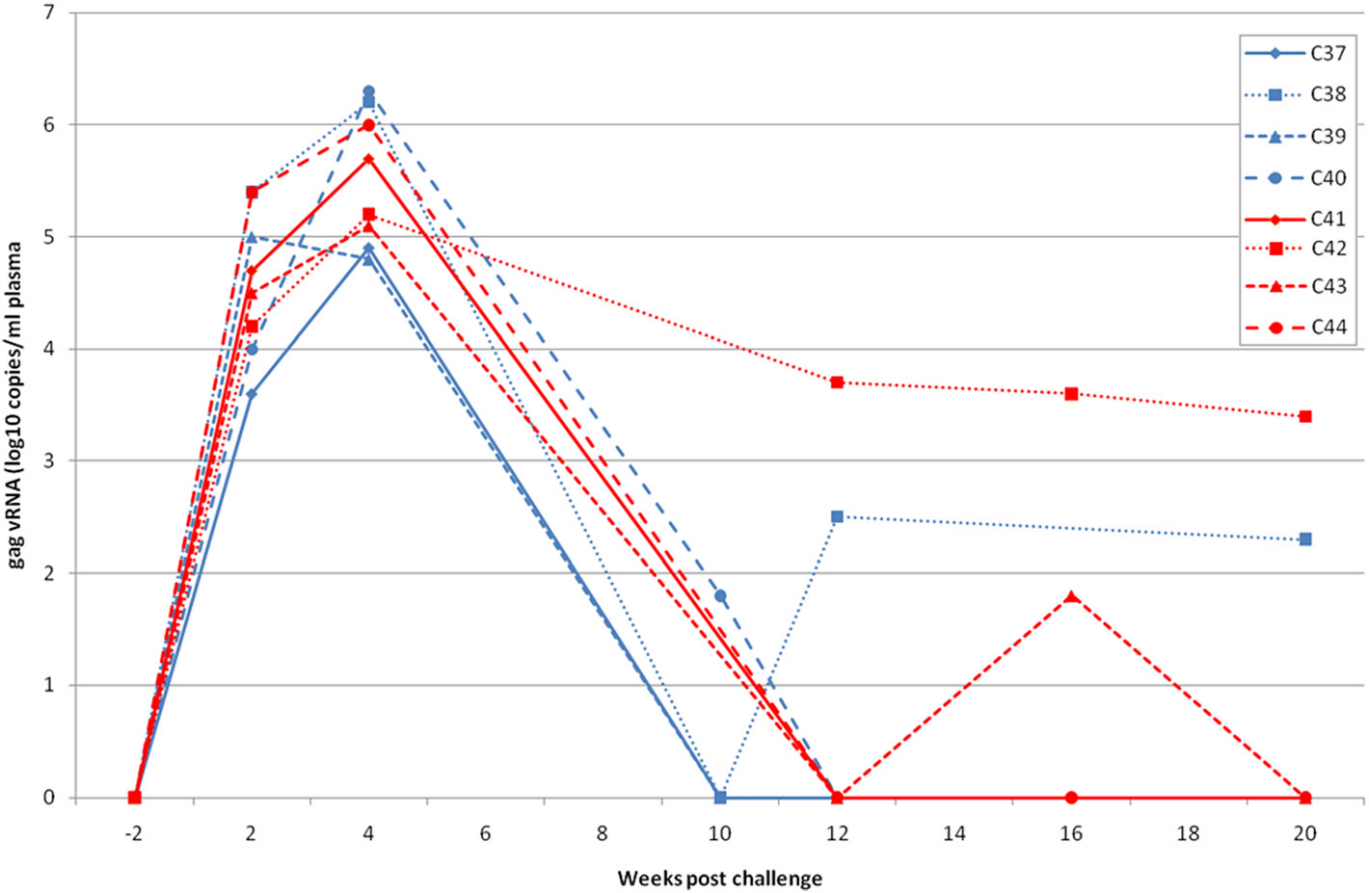
**Viral RNA levels in macaques given immune serum (C37-40; group E: blue lines) and contemporaneous controls (C41-44; group F: red lines) after challenge with 50MID**_**50**_**SHIV**_**sbg**_**and followed for twenty weeks.**

Serum collected from the 8 vaccinees (group A and B; A71-A78) that received the same vaccine protocol on multiple occasions between the 3^rd^ and 8^th^ immunisations with HIV-1_W61D_ rgp120 was pooled and 25ml/kg transferred into each of 4 naïve macaques (group E; C37-C40; Table 1). Twenty four hours later, they were challenged intravenously with 50 MID_50_ SHIV_sbg_ along with 4 naïve controls (group F: C41-44: Table 1). At the time of challenge, binding antibodies against HIV-1_W61D_ rgp120 (mean log_10_ titre 4.05+/−0.19) and HIV-1 IIIB rgp120 (mean log_10_ titre 3.75+/− 0.17; Table 4) were detectable in the recipients. In addition, neutralising activity against the homologous SHIV_W61D_ (mean log_10_ titre 2.4+/− 0.06) but not against the challenge virus SHIV_sbg_ (Table 4) was detected in these individuals. At 2 weeks after challenge, SHIV_sbg_ was detectable in all 4 controls as well as all 4 recipients of immune serum (Table 5). The peak vRNA loads at 2 weeks in naïve challenge controls (log_10_ 4.70 +/− 0.49) were not significantly different from recipients of immune serum (log_10_ 4.51+/0.84; Figure 2). Furthermore, the persistence of virus in blood, assessed by virus recovery and DNA PCR, was similar for the two groups of macaques (Table 5).

**Table 4:** **Serum transfer and challenge with SHIV**_**sbg**_

**GROUP**	**ID NO.**	**VACCINE**	**SEROLOGICAL RESPONSES**			
			**Binding antibodies(log_10_ reciprocal titre)DOC^a^**		**Virus neutralisation activity (50% virus inhibition log 10 titre) DOC_a_**	
			**W61D rgp120**	**IIIB rgp120**	**SHIV**_**W61D**_	**SHIV**_**sbg**_
E	C37	Serum from group A	3.9	3.9	2.4	<1
	C38		3.9	3.9	2.4	<1
	C39		4.3	3.6	2.5	<1
	C40		4.1	3.6	2.5	<1

DOC = day of challenge.

^a^ = time of sampling.

**Table 5:** **Detection of virus following transfer of immune serum from W61D rgp120 immunised macaques and challenge with SHIV**_**sbg**_**(50 MID50 dose)**

**GROUP**	**ID NUMBER**	**TREATMENT**	**OUTCOME OF CHALLENGE (Weeks Post Challenge)**							
			**−2**		**2**		**4**	**9**	**10**	**20**
			**VI**	**VI**	VT*	**PCR**	**PCR**	**VI**	**VI**	**VI**
E	C37	50 ml pooled serum from A71, A75, A76, A78 i.p day −1	-	+	2.5	-	+	+	ND	-
	C38		-	+	2.0	+	+	+	ND	+
	C39		-	+	2.0	+	+	+	ND	-
	C40		-	+	1.5	+	+	+	ND	-
F	C41	None	-	ND	2.5	+	+	ND	-	-
	C42		-	ND	1.0	-	+	ND	-	-
	C43		-	ND	3.0	+	+	ND	-	-
	C44		-	ND	3.0	+	+	ND	+	-

VT* = Virus Titration; Number of SIV + ve cells per 10^6^ PBMC.

VI = Virus isolation by co-culture of 5 x 10^6^ PBMC with 10^5^ C8166 indicator cells.

PCR = Diagnostic DNA PCR specific for SIV gag.

ND = Assay not performed.

### Immunisation with HIV-1_W61D_ rgp120 does not protect against SHIV_SF33_ challenge

Four weeks after the final (8^th^) immunisation, macaques in group B were challenged intravenously with SHIV_SF33_[12] (Table 1). After challenge, SHIV_SF33_ was detected in all the vaccinated macaques (A72, A73, A74, A77) at 2 weeks by DNA PCR and/or RT-PCR of plasma (Table 6). It was also possible to re-isolate virus by co-culture of PBMC with C8166 indicator cells from all 4 vaccinated macaques 4 weeks after challenge (Table 6). From 8 weeks to 20 weeks, detection of virus in the blood became more sporadic.

**Table 6:** **Detection of virus following challenge with heterologous SHIV**_**SF33**_**(50 MID50 dose)**

**GROUP**	**ID NO.**	**VACCINE**	**OUTCOME OF CHALLENGE(Weeks Post Challenge)**								
			**−4**	**2**	**4**	**8**		**12**		**20**	
			**VI**	**PCR**	**VI**	**VI**	**PCR**	**VI**	**PCR**	**VI**	
B	A72	30 μg W61D rgp120/AS02_A_ i.m	-	+	+	ND	+	-	+	-	
	A73		-	+	+	ND	+	-	+	-	
	A74		-	-	+	ND	-	+	+	-	
	A77		-	+	+	ND	+	+	+	-	
D	B238	AS02_A_ i.m	-	+	+	-	+	-	+	-	
	B239										
	B240		-	+	+	-	+	-	+	-	
	B241		-	-	+	-	+	-	+	-	
			-	+	+	-	-	+	+	-	

VI = Virus isolation by co-culture of 5 x 10^6^ PBMC with 10^5^ C8166 indicator cells.

PCR = Diagnostic DNA PCR specific for SIV gag.

ND = Assay not performed.

**Figure 3:**
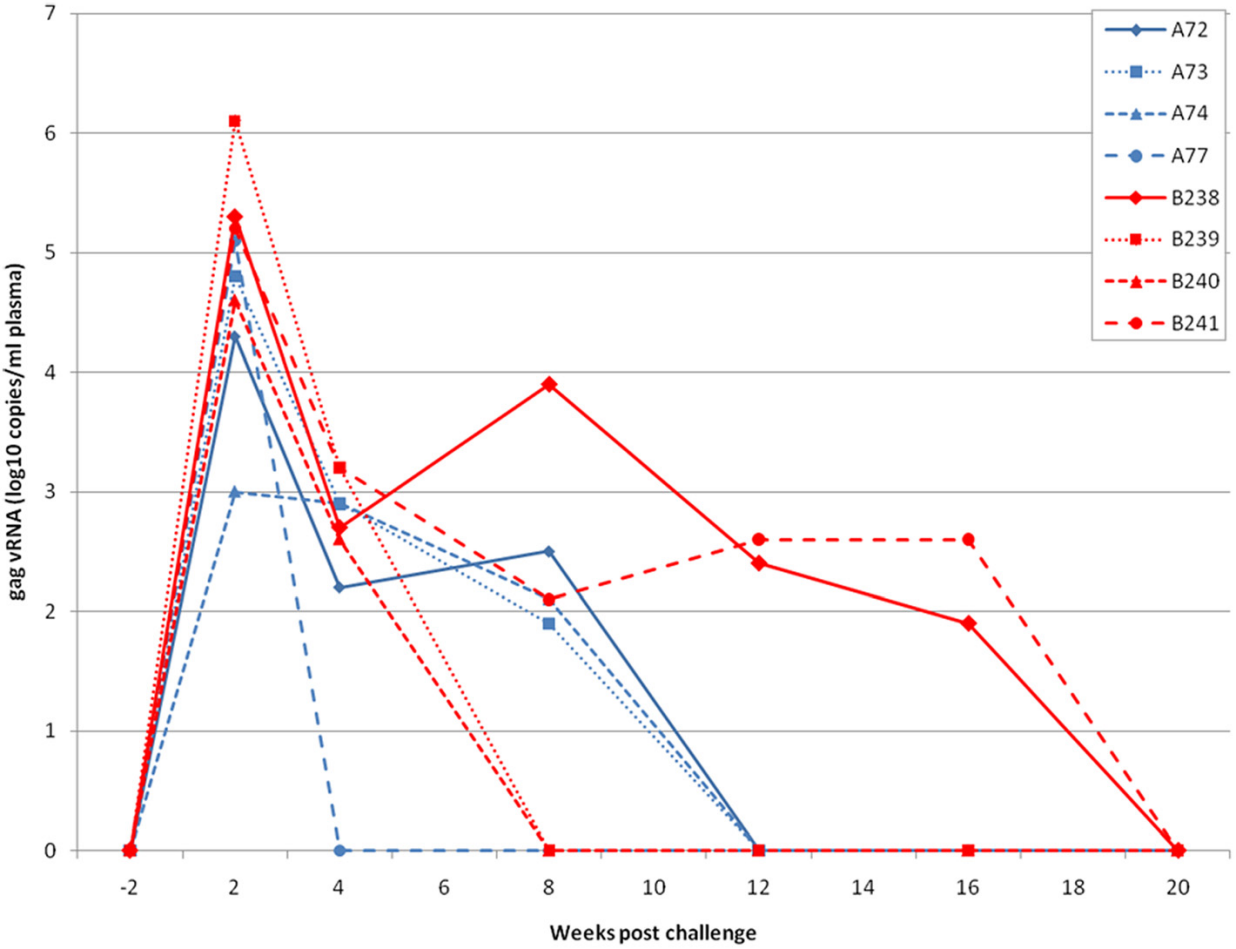
**Viral RNA levels in vaccinated macaques (A72, A73, A74 and A78; group B: blue lines) and contemporaneous controls given ASO2**_**A**_**adjuvant only (B238-241; group D: red lines) after challenge with 50MID**_**50**_**SHIV**_**SF33**_**and followed for twenty weeks.**

At 2 weeks after challenge the log_10_ mean viral load (4.23± 0.94) in vaccinees was not significantly different from unvaccinated controls (5.3 ± 0.62; Figure 3). Maximal vRNA loads were detected in plasma of vaccinees at 2 weeks. The vRNA levels declined thereafter to undetectable levels at week 12. At 2 and 8 weeks after challenge, animal A74 alone was negative by DNA PCR but became positive at week 12. All vaccinees were found to be positive by virus co-culture assay at 4 weeks but at 12 weeks only two macaques (A74 and A77) were positive. At 20 weeks, virus or vRNA could not be recovered from any vaccinee.

All of the unvaccinated controls (group D: Table 1) became infected by 4 weeks by virus co-culture assay and therefore mirrored the situation observed for the vaccinees (Table 6). No virus was detected within this group at 20 weeks after challenge by co-culture. All controls were positive by DNA PCR at 12 weeks. The vRNA levels also showed a similar profile to that of the vaccinees with a peak viraemia at 2 weeks followed by a decline to undetectable levels by 20 weeks in all animals (Figure 3).

### MHC typing of cynomolgus macaques

**Figure 4:**
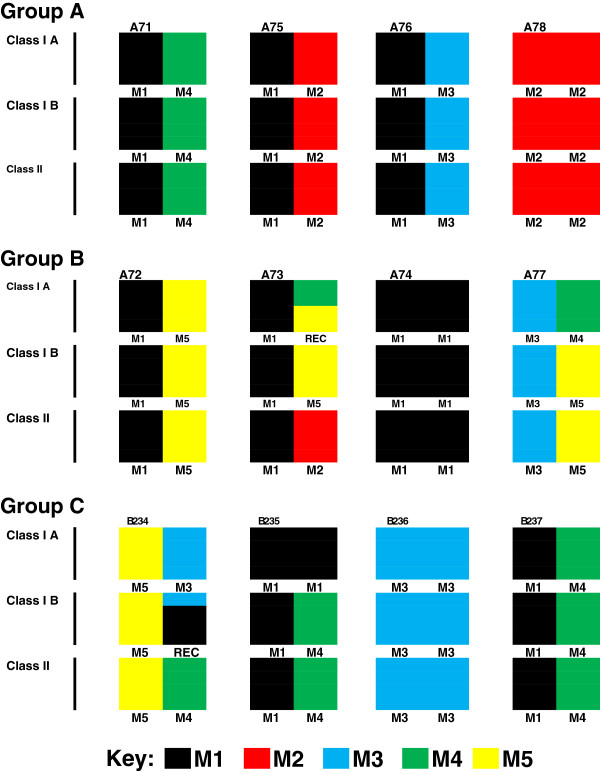
**MHC haplotypes of study animals were determined by microsatellite analysis and haplotypes with recombinants resolved by allele-specific PCR.** Intact haplotypes, M1–M5, have been previously identified in Mauritian cynomolgus macaques (37, 37) are designated by different colours (see key) for each of the animals used in groups **A**, **B** and **C.**

The results of MHC typing of cynomolgus macaque in groups A, B and C are shown in Figure 4. Whilst one macaque in each of groups A, B and C is homozygous for M2, M1 and M3 haplotypes, respectively, the remaining macaques were simple heterozygotes or possessed a simple recombination across MHC class I haplotypes. Across MHC class II haplotypes macaque A71 in group A was a match for B235 and B237 in group C whereas macaque A75 in group A was a match for A73 in group B. In a follow up study to analyse CD4+ T cell responses macaque G19, which was heterozygous for M1/M3, and G21, which was heterozygous for M1/M4, were selected as they were MHC haplotype matches for group A macaques A76 and A71, respectively.

### Immunisation with HIV-1_W61D_ rgp120 elicits potent T cell proliferative responses

In the initial challenge studies, insufficient samples were taken for analysis of cell-mediated immunity as we believed that protection would be antibody mediated as per homologous protection. When this proved not to be the case for heterologous protection, a retrospective study was undertaken to analyse cell-mediated immune responses. For this study, we selected two macaques, G19 and G21 (group G: Table 1), that were MHC matched with two animals from group A vaccinees, A76 (M1/M3 haplotype) and A71 (M1/M4 haplotype) respectively, which had resisted SHIV_sbg_ challenge (Table 3, Figure 4).

For analysis of T cell proliferative responses, peptides pools were selected on the basis of sequence homology of at least 9 consecutive amino acid residues, sufficient to constitute a shared T cell epitope, between W61D and IIIB or SF33 HIV-1 envelope sequences. Hereafter, homology is referred to in terms of potential shared T cell epitopes. Peptides across variable loops 1, 2, 3 and 4 showed little homology between W61D, IIIB and SF33 sequences and were divided into 3 pools that approximately covered V1/V2, V3 and V4 regions for each envelope sequence (Table 7). Five peptides across C1 and two within C2 that showed homology between W61D, IIIB and SF33 sequences formed the C1 peptide pool (Table 7). Four further peptides within C2 that showed homology between W61D and IIIB but not SF33 formed the C2 pool and this was compared with the corresponding SF33 C2 pool (Table 7). These four C2 peptides with W61D/IIIB homology were tested individually against corresponding SF33 C2 peptides (Table 7). Two peptides across C3 showed homology between W61D and SF33 but not IIIB formed the C3 pool (Table 7).

**Table 7:** Composition of HIV-1 envelope peptide pools tested

**Peptide Pools**	**W61D**	**IIIB**	**SF33**
**V1/V2 Pool**	ARP7035.6-17	ARP740.6-16	ARP7117.6-16
**V3 Pool**	ARP7035.22, 25-33	ARP740.21, 24-33	ARP7117.21, 24-33
**V4 Pool**	ARP7035.34-38, 41-48	ARP740.33-38, 41-47	ARP7117.33-38,41-46
**C1 Pool**	ARP7035.1-5, 20, 21	nt	nt
**C2 Pool**	ARP7035.18, 19, 23, 24	nt	ARP7117.17, 18, 22, 23
**C3 Pool**	ARP7035.39, 40	nt	nt

nt = not tested.

20mer HIV-1 env gp120 peptides with 10mer overlap from CFAR, NIBSC, UK.

**Figure 5:**
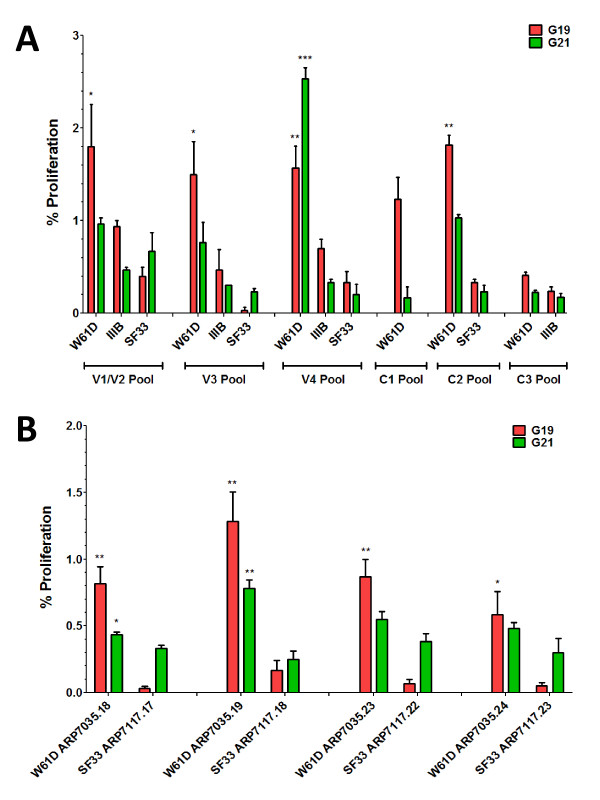
**Proliferative responses of HIV-1 rg120 W61D vaccinee PBMC to HIV-1 env gp120 peptide pools.** Proliferation was measured by flow cytometry using a CFSE dye dilution assay reporting the percentage of cells that have undergone at least two cell divisions. Assays were performed in triplicate and the mean response ± SEM are shown. Responses to the vaccine isolate W61D peptide pools were compared to heterologous IIIB and SF33 isolates. Panel **A** shows the response to peptide pools covering variable regions V1/V2, V3 and V4. The C1 pool contains peptides with homology across all three isolates. The C2 pool contains peptides with homology between W61D and IIIB. The C3 pool contains peptides with homology between W61D and SF33. Panel **B** compares the mean responses of the 4 peptides that comprise the C2 pool, with homology between W61D and IIIB, with the corresponding non-homologous SF33 peptides, individually. A strong response to the W61D peptide ARP7035.19 is common to both G19 and G21which is lost when the corresponding SF33 peptide ARP7117.18 is substituted.

All proliferative T cell responses detected against peptide pools were mostly (>90%) CD4+ T cell responses (data not shown). G19 made a broad proliferative T cell response that was significant against W61D V1/V2, V3 and V4 peptide pools, compared with corresponding SF33 peptide pools (Figure 5A). By contrast, the G21 response was strongly focused on and highly significant for the V4 peptide pool (Figure 5A). Responses against IIIB and SF33 V1/V2, V3 and V4 peptide pools were greatly reduced by comparison with W61D responses. Only G19 made a proliferative response against the consensus C1 peptide pool (Figure 5A). Both G19 and G21 made significant proliferative responses against the C2 peptide pool compared with the corresponding SF33 peptide pool response (Figure 5B). Neither G19 nor G21 made a significant proliferative response against the C3 peptide pool (Figure 5B). When C2 peptides were tested individually, G19 made a significant proliferative response against all four peptides within the pool, compared with the corresponding SF33 peptides, with the strongest response directed against peptide ARP7035.19 (Figure 5B; Additional file 1: Table S 1). G21 made significant proliferative responses against two of the four peptides within the C2 pool, compared with the corresponding SF33 peptides. The strongest response was also directed against peptide ARP7035.19 (Figure 5B; Table S1). G21 also made a significant proliferative response against ARP7035.18, which overlaps ARP7035.19 by 10 amino acids (Figure 5B; Table S1). Comparison of sequences across the region covered by peptide ARP7035.19 revealed an identical sequence for W61D and IIIB with only a single amino acid change at position 213 (A to T) for the equivalent SF33 peptides ARP7117.18 that may account for absence of a T cell response (Additional file 1: Table S1).

## Discussion and Conclusions

A consensus scientific viewpoint has emerged that future licensed AIDS vaccines should include HIV-1 envelope protein as a component [23-25]. A recent macaque study also highlighted the importance of including envelope protein in the vaccine formulation to achieve protection from infection [19]. The challenge is to formulate envelope based vaccines that confer broad protection against this hypervariable viral antigen. In order to achieve this, we need to define in detail, the protective immune responses that envelope based vaccines need to elicit. Whilst this information could be ascertained from Phase 3 clinical trials, the size and cost of these trials is such that testing in appropriate animal models is attractive, if they are able to provide pertinent information. This HIV-1_W61D_ envelope component has been formulated and tested in Phase 1 clinical trials either alone [26] or in combination with other recombinant HIV-1 protein antigens [27,28]. This study was designed to evaluate this same vaccine to protect against challenge with viruses that expressed a heterologous B clade HIV-1 envelope. We observed that protection against infection by a chimaeric virus is feasible even in the absence of a detectable cross neutralising antibody response against the envelope of the challenge virus. Protection appears to correlate with cell mediated immunity, specifically CD4+ T cell responses against an epitope within a conserved domain (C2) of HIV-1 gp120 (with the caveat that this was observed in a study of only 2 animals).

In previous studies, it was shown that immunisation with the same candidate AIDS vaccine based upon HIV-1_W61D_ rgp120 formulated in AS02_A_ adjuvant protects macaques against intravenous challenge with SHIV_W61D_ expressing the homologous envelope [10] and that immune serum collected from immunised macaques could transfer this protection (Almond et al., manuscript in prep). In previous observations, increasing the number of vaccinations above three doses enhanced the neutralising antibody titres whilst the binding antibody titres remained constant (Almond et al., manuscript in prep). This suggests that the additional immunisations result in qualitative changes in the neutralising antibody responses. These responses appeared to be focussed primarily against variable regions of the envelope, therefore limiting the potential breadth of protection. In our initial study designed to interrogate the breadth of protection possible with HIV-1 envelope based vaccines, no cross protection was observed, when a three dose vaccine schedule had been employed [9]. However, other studies have indicated that protection against heterologous SHIV was possible, when the heterologous SHIV challenge occurred as a follow up after vaccine recipients had resisted homologous SHIV challenge [12] (MP, NA, RS unpublished observations). Nevertheless, there existed the possibility that the improved breadth of protection of envelope based vaccines was due to exposure to other less variable viral antigens during the initial homologous challenge, and this led to a broadening of the protection against heterologous viruses [12]. Our study was designed to establish the breadth of protection after the extended vaccine regimen without this confounding issue of virus re-challenge.

In the first challenge study, the eight immunisation protocol appeared to protect the vaccinees from detectable infection with SHIV_sbg_. PCR methods for virus detection failed to detect any direct evidence of the viral DNA or RNA. However, although the virus stock had been titrated in cynomolgus macaques previously, the 10 MID_50_ challenge dose only infected three of four naive challenge controls. It is unclear whether this was a result of incorrect dilution of the virus challenge stock or a stochastic event due to a probability by Poisson distribution that a low dose inoculum will not contain a single infectious agent. We have used this stock of virus in two previous studies at this dose and have infected successfully all of eight naive challenge controls [29]. Using these controls there are 11/12 animals that became infected with the 10MID50 dose compared with 0/4 vaccinated animals which by Fischer’s exact test returns a statistically significant difference of p = 0.003.

To investigate the role of antibody in protection, serum from vaccinated macaques was transferred into naive recipients that were then challenged with the heterologous SHIV_sbg_ challenge. None of these animals resisted the virus challenge despite the serum being derived from macaques that had resisted the same SHIV challenge. Moreover, neutralising activity against the homologous virus (SHIV_W61D_) in the serum remained relatively high at 2.4 log_10_ 50% viral inhibition titre (Table 2). These data contrast with the outcome of our previous passive transfer studies where smaller volumes of immune serum collected from HIV-1 rgp120 immunised macaques have protected naïve recipients even when challenged with 10–50 MID_50_ of a SHIV expressing an envelope homologous with the vaccine (MP, NA, RS unpublished observations). It is possible that the failure to protect was due to the higher (50MID_50_) dose challenge used after the serum transfer compared with the prior challenge (10MID_50_) after vaccination. The higher dose was employed to ensure that all the control animals were infected which is necessary to achieve statistical significance. However if this 5 fold higher virus dose was sufficient to overcome the saturation point of the protective antibodies in the transferred serum then differences in the kinetics of virus replication (such as the peak virus load) in naive challenge controls and recipients of immune serum may be anticipated; this was not the case (Figures 1 and 2) as has been reported previously for SHIV challenge [30] . Furthermore, the peak viral loads in the naive control animals at 2 weeks receiving either the 10MID_50_ dose (group C) or the 50MID_50_ dose (Group F) were not significantly different (Student’s *t* test p = 0.38).

Unlike the initial challenge with SHIV_sbg_, eight immunisations with HIV-1_W61D_ rgp120 did not protect against challenge with SHIV_SF33_. Following challenge, the kinetics of virus replication was not significantly different between vaccinates and naive challenge controls. Perhaps this was not surprising since, at the time of challenge, only very limited cross neutralisation was detectable in the serum. Indeed, the additional immunisations did not result in any improvement of vaccine protection, compared with our previous study, using this challenge virus [9].

The demonstration of protection in the absence of detectable virus cross-neutralising antibodies and the lack of protection by serum transfer suggests that other anti-HIV-1 envelope responses are required to protect against heterologous SHIV challenge. It has been reported that this vaccine is able to elicit CD4+ but not CD8+ T cell responses in vaccine trial volunteers [27,28] and this has been corroborated in another study in macaques (Almond et al., manuscript in prep). We decided therefore to investigate the specificity of CD4+ T cell responses using overlapping HIV-1 env peptides for both vaccine and challenge viruses to determine if there was any correlation with protection. Our rationale was that any correlate of heterologous protection must be conserved between the vaccine and challenge virus sequence where there was protection (SHIV_sbg_) and not where there was absence of protection (SHIV_SF33_). Furthermore, the conserved T cell response must be preserved in macaques with different MHC haplotypes as all group A vaccinees were protected. Notably the animals in this group all possessed at least one copy of the M1, M2 or M3 haplotype; each of these haplotypes shares common class IA alleles Mafa-A1*063:01/02Mafa-A2*05:01/11 and Mafa-A4*01:01, but class II allele sharing is limited [31,32].

Analysis of CD4+ T cell proliferative responses against variable regions of HIV-1_W61D_ env revealed strong but differential responses in both macaques that may be due to their different MHC haplotypes. The M1/M3 haplotype of G19 appeared to favour a broad CD4+ T cell response whilst the M1/M4 haplotype of G21 favoured a strongly focused anti-V4 response. It could be concluded from this result that CD4+ T cell proliferative responses against V4 are a correlate of protection, since it is common to both challenge viruses. However as all anti-V4 responses were lost when the corresponding IIIB and SF33 peptide pools were used to restimulate cells, then it could not be a correlate of heterologous protection. Similar sequence divergence between the three challenge viruses across all variable regions prevented CD4+ cell responses against these regions of envelope being potential correlates of vaccine protection.

By contrast, a number of peptides spanning the more conserved regions of HIV-1 env [33] proved more interesting. The highly conserved C1 region did not exhibit sufficient sequence variation between the vaccine and challenge SHIVs to account for the differential outcome of SHIV_sbg_ and SHIV_SF33_ challenges. For conserved region three, only one of the two MHC typed macaques made detectable CD4+ responses even against peptide based on the HIV-1_W61D_ envelope homologous to the vaccine. This left just four peptides from conserved region two (C2) that were homologous between W61D and IIIB but heterologous for SF33. Both vaccinated macaques tested made significant CD4+ T cell responses to peptides based on the SHIV_W61D_ and SHIV_sbg_ C2 region and these were lost when the corresponding peptides from SHIV_SF33_ were used. Although there were differences between the precise epitope recognised by each macaques studies, which is not surprising since they were of different MHC type, both responses mapped to a region where the sequence of SHIV_sbg_ and SHIV_W61D_ envelopes are the same and distinct from SHIV_SF33_.

Although it would appear unexpected that non-antibody mediated protection elicited by HIV-1 envelope vaccines could map to a restricted region of the conserved region 2 of this protein covered by a region between two and four peptides, previously published data on cross protection would appear supportive. Mooij *et al*. [12] reported that, following vaccination with this or closely related vaccine formulations of HIV-1_W61D_ rgp120, they observed protection against challenge with both heterologous SHIV_SF13_ and SHIV_HAN-2_. Remarkably, sequence analysis of gp120 reveals sequence homology across the C2 region between these SHIVs and HIV-1_W61D_ and HIV-1 IIIB (Additional file 1: Table S1). By contrast, Voss *et al*. [15] reported that a closely related vaccine incorporating the same envelope component did not protect against SHIV_89.6p_ infection. The envelope of SHIV_89.6p_ exhibits two amino acid differences across the peptide ARP7035.19 from the vaccine, although these differences are not identical to those found in SF33 (Additional file 1: Table S1).

Although we only used a 3 dose schedule for the follow up study of 2 animals (group D) rather than an 8 dose schedule used for the challenge studies, we predict that this would result in a protective response against SHIV_sbg_ based on the study by Mooij *et al*. [12] where heterologous protection was observed with the same vaccine formulation and a 6 dose schedule. It is likely that the cross reactive cell mediated responses we observe after 3 immunisations would still be dominant after 8 immunisations and therefore afford protective status but there may be a further broadening and/or maturation of responses to the conserved regions.

Whether these preliminary observations are coincidental is uncertain at the moment. Nevertheless, investigating whether this apparent correlate defines a novel mechanism of vaccine protection conferred by HIV-1 envelope vaccines in the absence of cross neutralising antibodies is testable using the Mauritian derived cynomolgus macaque (MCM). Protection against infection in the absence of neutralising antibodies has been observed for influenza [34] and SIV [19,20]. The mechanism is likely to be through T helper subsets, specifically effector memory cells [20] and therefore the adjuvant (such as AS02) used in vaccine formulations will be important in driving the immune response to a protective or non-protective T helper bias; for example in protective vaccines for malaria [35].

The limited genetic diversity at the MHC level of MCM due to a small founder population, permits group sizes that are immuno-genetically defined across both Class I and Class II regions of the MHC. A more detailed analysis of cellular immunity is therefore warranted to investigate the response to the C2 peptide identified in this study, in adequately sized groups of vaccinated macaques; challenge of these groups with viruses selected by their sequence across C2 would generate incontrovertible data whether the CD4+ cellular responses identified in this study can contribute to the protection in the absence of neutralising antibodies. If so then it would open up new avenues for the development of novel broadly protective vaccines against HIV/AIDS.

## Methods

### Animals

Purpose bred juvenile Mauritian cynomolgus macaques (*Macaca fascicularis*) obtained from a simian retrovirus free colony, were housed and maintained in accordance with Home Office guidelines for the care and maintenance of primates. Animals were sedated with Ketamine before immunisation, venepuncture or clinical examination.

### Vaccination with HIV-1_W61D_ rg120

The recombinant HIV-1_W61D_ rgp120 antigen was engineered from an envelope molecular clone derived from the Dutch clinical HIV-1 isolate ACH320 [36] and expressed in a mammalian expression system using Chinese hamster ovary cells. The rgp120 antigen (100 μg) was reconstituted with 0.5 ml of AS02_A_ (GlaxoSmithKline Biologicals; o/w emulsion-based Aduvant System containing 50 μg MPL and 50 μg QS21 [Antigenics, New York, USA]) prior to vaccination. In immunisation protocols, control animals were administered AS02_A_ Adjuvant System without antigen.

A group of four macaques (group A, A71, A75, A76, A78) was immunised intramuscularly on 8 occasions with 100 μg AS02_A_–adjuvanted HIV-1_W61D_ rgp120 at 0, 4, 12, 20, 28, 36, 44 and 86 weeks. A group of four (naive) macaques (group C, B234-B237) was used as challenge controls. Group A was challenged with 10MID_50_ SHIV_sbg_ 2 weeks after the last immunisation simultaneously with group C animals.

Similarly, a group of four macaques (group B; A72, A73, A74, A77) was immunised contemporaneously as for group A. A group of naive macaques (group D; B238-241) was used as challenge controls. Groups B and D were challenged simultaneously with 50MID_50_ SHIV_SF33_ 2 weeks after the last immunisation.

A group of two macaques (group G; G19 and G21) was immunised with the same vaccine formulation as for groups A and B but on 3 occasions only at 0, 4 and 8 weeks (Table 1) for retrospective analysis of cell mediated responses.

### Serum transfer

Immune sera were collected and pooled from eight macaques between immunisations 3 and 8 at weeks 30, 32, 35, 37, 42, 46, 48 and 50 before challenge. Four naïve macaques (Group E; C37-C40) received 25 ml/kg of pooled immune serum by intra-peritoneal injection. Four controls (Group F; C41-C44), received an equivalent volume of normal saline. Twenty four hours after transfer of serum or saline, all recipients were challenge with 50 MID_50_ SHIV_sbg_ intravenously.

### Heterologous SHIV challenge

The stock of SHIV_sbg_ used throughout was as previously described [21]. SHIV_sbg_ was prepared on macaque PBMC and had a titre of 1.9 x 10^5^ MID_50_/ml in *M. fascicularis*. The challenge dose of 10MID_50_ was given intravenously. An intravenous dose of 50 MID_50_ was used for serum transfer studies. SHIV_SF33_[13] was grown in macaque PBMC and had a titre of 1 x 10^3^ MID_50_ in *M. fascicularis*[9]. The challenge dose was 50 MID_50_ given intravenously.

### Detection of virus following challenge

The outcome of virus challenge was evaluated for twenty weeks after which studies were terminated. Evidence of virus infection in the macaques after challenge was based upon the recovery of virus either a) by co-culture of 5 x 10^6^ Ficoll purified monocytes with C8166 indicator cells as described previously [9] b) detection and quantification of viral RNA in plasma following real-time qRT-PCR mediated amplification of SIV gag sequences as described previously [37]. The lower limit of detection of the assay is 50 SIV RNA copies/ml or c) detection of proviral DNA by qDNA real-time PCR amplification was performed as described previously [37].

### Measurement of humoral immune responses

Binding antibodies were detected in plasma by ELISA using recombinant HIV-1_W61D_ rgp120 (EVA 648) and HIV-1 IIIB (EVA607) supplied by the Centralised Facility for AIDS Reagents (CFAR, NIBSC). Binding antibodies were detected using goat anti-human Ig coupled to horseradish peroxidase and end-point titre calculated as described previously [38]. Neutralising antibody activity was determined by a single cell infectivity assay using an indicator cell line Tzm-Bl an adapted method of Wei *et al*. [39]. The test serum was serially titrated in doubling dilutions in DMEM with 10% v/v FCS in 96 well microtitre plates in triplicate. 50 μl of 2 TCID_50_ concentration of virus was added to all wells and the antibody/virus mixture incubated for 45 minutes. The indicator cell line TZM-bl which expresses β-galactosidase and luciferase on infection with virus was added to the wells and the plate incubated at 37 °C for 48 hours. Following incubation, all the supernatant was aspirated and the cells lysed with 5% NP40. An aliquot of the lysate was transferred into an empty microtitre plate and β-gal substrate added (Novagen β-red β-galactosidase kit). The colour development was stopped with the kit stop solution and the absorbance read at 590 nm using an ELISA plate reader.

Viruses tested by neutralisation assay were SHIV_W61D_[27], SHIV-4 [40], SHIV_sbg_[21] and SHIV_SF33_[13].

### Measurement of cell-mediated immune responses

MHC haplotypes were defined by microsatellite analysis, with resolution of recombinant haplotypes by allele-specific polymerase chain reaction (PCR), to report the six most common Mauritian cynomolgus macaques MHC haplotypes, or simple recombinants thereof as previously described [41,42].

PBMC from immunised cynomolgus macaques G19 and G21 group G), isolated from the heparinised blood by Percoll density gradient separation (GE Healthcare Bio-Sciences AB, Uppsala, Sweden), were resuspended in RPMI 1640 culture medium (Sigma-Aldrich, Poole, UK) supplemented with 10% foetal calf serum, L-glutamine and antibiotics Penicillin and Streptomycin (Invitrogen Ltd, Paisley, UK). For measurement of lymphoproliferative responses PBMCs were labelled with 5, 6-carboxyfluorescien diacetate succinimdyl ester (CFSE; Invitrogen Ltd, Paisley, UK) according to manufacturer’s protocols. CFSE-labelled cells were incubated in 96-well, U bottom culture plate (Thermo Fisher Scientific, Loughborough, UK) at a density of 2x10^5^ cell per well in triplicate. PBMCs were stimulated with 20mer HIV-1 envelope peptides with a 10 amino acid overlap (CFAR, NIBSC, UK) at a concentration of 1 μg/ml/peptide for pools and at 5 μg/ml for single peptides (Table 7). Cultures were maintained at 37°C in a 5% CO_2_ incubator for 5 days. To confirm the phenotype of proliferating cells PBMC were labelled with APC-Cy7 conjugated monoclonal anti-CD4 (Biolegend Ltd, Cambridge, UK) for 20 min at room temperature. Samples were analyzed on FACS CantoII (Becton Dickinson, Oxford, UK). From each sample a minimum of 100,000 events was collected and analysed using FACSDiva software. Proliferation was reported as the percentage of cells that had undergone 2 or more rounds of cell division.

### Statistical analysis

For comparison of peptide pools one-way ANOVA with Bonferroni’s multiple comparison test was employed to assess differences between responses to W61D, IIIB and SF33. For comparison of responses to single peptides a Mann–Whitney *U* test was employed to assess differences between W61D/IIIB and SF33. *P* values <0.05 were considered significant (*), <0.005 highly significant (**) and <0.0001 very significant (***) using the Graph Pad Prism 5 software.

## Competing interests

G. Voss is an employee of GSK, N. Mathy was an employee of GSK at the time of the study. Authors affiliated with NIBSC declare that they have no competing interests.

## Author’s contributions

MP, RS, NA, NB, JS, NM, DF and GV conceived and designed the experiments; BL, RH, LDa, LDu, WE, JH, CH, MH, RQP, EM performed the experiments; MP, RS, BL, NA, NB, NR analysed the data; MP, NA and RS wrote the paper; all authors read and approved the final document.

## Supplementary Material

Additional file 1 **Table S1.** Comparison Of C2 HIV-1 envelope peptide sequences tested. Click here for file
