# Supplementary material for: Heterologous protection elicited by candidate monomeric recombinant HIV-1 gp120 vaccine in the absence of cross neutralising antibodies in a macaque model
Source: Retrovirology. 2012 Jul 16;9:56. doi: 10.1186/1742-4690-9-56 (PMC3418562; doi:10.1186/1742-4690-9-56)
Supplement: Additional file 1 — Table S1. Comparison Of C2 HIV-1 envelope peptide sequences tested. [file 1742-4690-9-56-S1.doc]

**TABLE S1: COMPARISON OF C2 HIV-1 ENVELOPE PEPTIDE SEQUENCES TESTED**

**201 211 221 231 241 251 261 271**

**W61D NFRLIHCNSSVMTQACPKVSFEPIPIHYCAPAGFAILKCNNKTFDGKGLCTDVSTVQCTHGIRPVVSTQLLLNGSLAEEEVV**

**IIIB SY---S--T--I--------------------------------N-T-P--N---------------------------D--**

**SF33 SY---------I--T---------------------------K-S---Q--N----------K-------------------**

**89.6P KY---S--T--I---------Q-------V--------------N-S-P--N---------------------------DI-**

**HAN2 SYM-----R--I----------------------------D-K-N---P-KN--------------------------K---**

**SF13 -Y------R--I--------------------------------N---P--N----------K-------------------**

**W61D DRLIHCNSSVMTQACPKVSFE:ARP7035.18 VSTVQCTHGIRPVVSTQLLL:ARP7035.23**

**SF33 SYRLIHCNSSVITQTCPKVS:ARP7117.17 TNVSTVQCTHGIKPVVSTQ:ARP7117.22**

**W61D TQACPKVSFEPIPIHYCAPA:ARP7035.19 RPVVSTQLLLNGSLAEEEVV:ARP7035.24**

**SF33 VITQTCPKVSFEPIPIHYCA:ARP7117.18 GIKPVVSTQLLLNGSLAEEE:ARP7117.23**
